# Polyglot Search Translator

**DOI:** 10.29173/jchla29600

**Published:** 2022-04-01

**Authors:** Janice Y. Kung

**Affiliations:** Public Services Librarian, University of Alberta, Edmonton AB, Canada

**Product:** Polyglot Search Translator.

**URL:** https://sr-accelerator.com/#/polyglot

**Intended audience:** MEDLINE searchers of any experience level.

## Product Description

The Institute for Evidence-Based Healthcare (IEBH) at Bond University developed a suite of tools called the Systematic Review Accelerator [1]. All the tools featured on the website are freely available to users. The Polyglot Search Translator (the Polyglot) is one of the featured tools, designed to translate search strings across databases to help researchers save time when running searches for systematic reviews. The Polyglot translates either a PubMed or Ovid MEDLINE search string into several database platforms including PubMed, Ovid MEDLINE, Cochrane Library, Embase (via Elsevier), Ovid Embase, Web of Science (simple and advanced searches), Scopus (basic and advanced searches), PsycInfo (via Ovid), ProQuest Health and Medical, SPORTDiscus, and PubMed Expanded. It also provides an option for a lexicalTreeJSON output. Users may create a free account but it is not required to use the Polyglot.

## Intended Users

The Polyglot is intended for systematic review researchers (including librarians and other information professionals) who have familiarity with developing and executing searches so that they may expedite the process of translating one search strategy (either PubMed or Ovid MEDLINE) into other databases.

## Special Features

There is one main search query box for users to copy and paste their search strategy. The icons below perform specific actions:

**Table:** 

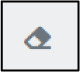	Clears the search query box
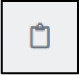	Copies the search string to a clipboard
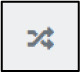	Shows a random example of a search string

**Fig. 1:**
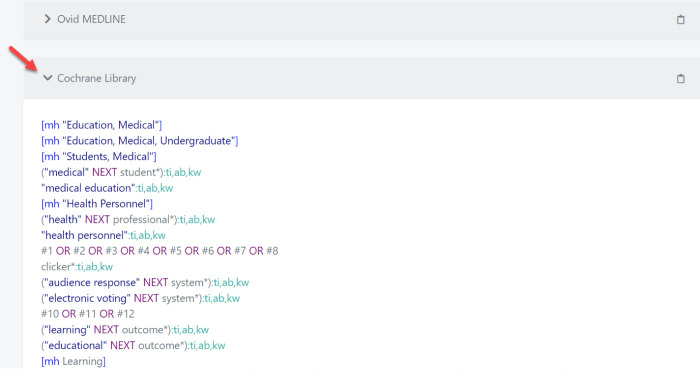
Screen Capture of how Translated Searches Appear for Cochrane Library

After copying and pasting a search string, scroll down to see the search translation across various databases and/or platforms by clicking on the arrow to expand each section (Figure 1). Translated searches may also be copied to a clipboard, using the same icon displayed on each database header.

There is a way to visualize the search strategy by clicking on “Open Query in SearchRefiner” for a network analysis of how the terms relate to one another and their citation count. Rather than copy/pasting the search strategy, users may opt to import the search strategy from a text file (.txt file). Polyglot colour codes different parts of the search to optimize readability. For instance, terms written in dark blue font indicate that there is a pop-up explanation or warning that the user should check to see if the translation was done properly. MeSH terms are generally highlighted in blue while terms in purple font represent boolean operators. Unrecognized characters are highlighted in yellow (e.g. wildcards).

One of the best features of the Polyglot is the ability to “take a numbered line search, and rather than translate each individual line, it can expand the line by line search string into a single search string.” This helps users save a lot of time so that they do not need to copy/paste the entire search strategy line by line into another database (see Figures 3 and 4).

**Fig. 2:**
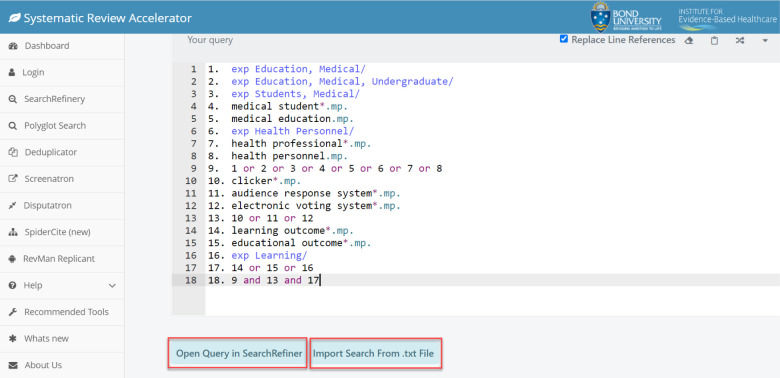
Additional Features Available such as SearchRefiner and Import Search From .txt. File

**Fig. 3:**
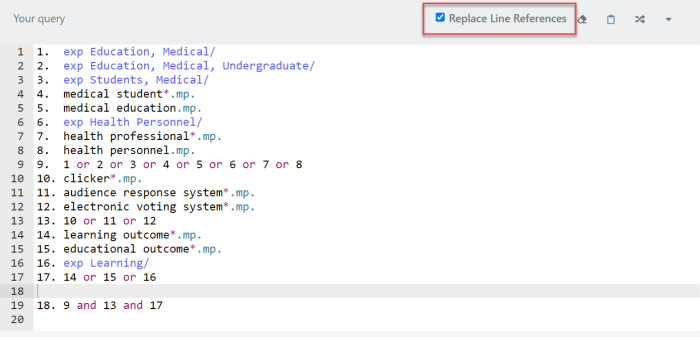
“Replace Line References” Translates the Search into a Single Search String

**Fig. 4:**
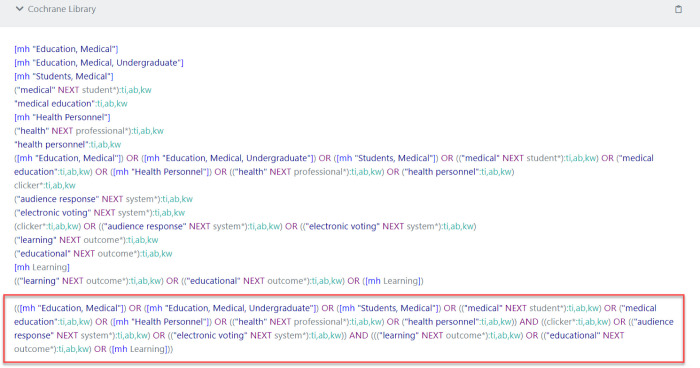
Sample Translated Search for Cochrane Library as One Search String

## Compatibility

The Polyglot Search Translator works well across different web browsers (Chrome, Firefox, Microsoft Edge, and Safari).

## Usability

Polyglot’s interface is user-friendly and simple in its design for a new user to quickly grasp how the tool functions. Even if users do not understand the icons available, they may hover their mouse over each icon to see the functionality. The Help page is also easy to find and navigate.

## Strengths

Polyglot is ideal for searches that are not too complicated and works well in translating searches to other database platforms. There is evidence to suggest that using the Polyglot helps searchers save time and reduces the mean number of errors in comparison to manual searching [2]. The help section is thorough and in-depth. It provides step-by-step instructions on how to use the tool.

## Weaknesses

Complicated searches that include frequency operators (e.g. hope.ab. /freq=2), wildcards, comments (i.e. text in square brackets → exp Pancreatic Neoplasms/su **[Surgery]**), or special limiter searches in MEDLINE (e.g. limit x to covid-19, where x represents the line number) do not translate accurately so it is imperative for users to understand the search process fully in order to make the proper corrections for each translated search. For example, hope.ab. /freq=2 translates as hope/ in Ovid Embase. In another example, the translated search for Cochrane Library will generate an error message if there are square brackets present in the original Ovid MEDLINE search.

One of the critical processes of developing searches is the iterative process that occurs when searching individual databases organically for additional terms. If users are not experienced searchers, they may take the search translations at face value and miss the process of discovering other relevant terms, when browsing subject headings as an example, or identifying certain quirks of databases that may compel the searcher to modify and optimize the search for particular databases. For example, Embase is known to over index its publications using Emtree terms, which lowers precision in article retrieval [3, 4]. Therefore, searchers are likely to build different searches in MEDLINE compared to Embase for drug-related systematic reviews.

## Comparison with Similar Products

Medline Transpose [5] has similar functionality to the Polyglot but it is not as comprehensive. It is also free but can only translate searches from PubMed to Ovid MEDLINE and vice versa. Users are recommended to use Chrome, Firefox, or Safari for best results. The copy and paste function is not as seamless in Medline Transpose. Normally when searches are copied from Ovid MEDLINE, the line references remain. Medline Transpose cannot translate properly if the numbers corresponding to each line are copied. This is not a limitation in the Polyglot since it has the feature, “Replace Line References.” The search documentation is very thorough and breaks down how the search is translated for each field code. Similar to the Polyglot, Medline Transpose is unable to translate complex searches that include wildcards or proximity operators since proximity operator searching is not a function enabled in PubMed.

## Conclusion

Polyglot is an excellent tool to help systematic review researchers build their searches within certain limitations. The searcher must have a very good grasp of search formulation and the search process so that translated searches may be revised, if needed. Even though it cannot fully translate all aspects of the search strategy, it may be used as an educational tool to demonstrate how to translate searches across different database platforms.
